# WilsonGen a comprehensive clinically annotated genomic variant resource for Wilson’s Disease

**DOI:** 10.1038/s41598-020-66099-2

**Published:** 2020-06-03

**Authors:** Mukesh Kumar, Utkarsh Gaharwar, Sangita Paul, Mukta Poojary, Kavita Pandhare, Vinod Scaria, Binukumar BK

**Affiliations:** 1 CSIR Institute of Genomics and Integrative Biology, Mathura Road, Delhi, 110 025 India; 2 Academy of Scientific and Innovative Research, CSIR-IGIB South Campus, Mathura Road, Delhi, India

**Keywords:** Computational biology and bioinformatics, Genetics, Diseases, Gastroenterology, Medical research, Molecular medicine, Neurology

## Abstract

Wilson disease (WD) is one of the most prevalent genetic diseases with an estimated global carrier frequency of 1 in 90 and a prevalence of 1 in 30,000. The disease owes its genesis to Kinnier Wilson who described the disease, and is caused by accumulation of Copper (Cu) in various organs including the liver, central nervous system, cornea, kidney, joints and cardiac muscle which contribute to the characteristic clinical features of WD. A number of studies have reported genetic variants in the ATP7B gene from diverse ethnic and geographical origins. The recent advent of next-generation sequencing approaches has also enabled the discovery of a large number of novel variants in the gene associated with the disease. Previous attempts have been made to compile the knowledgebase and spectrum of genetic variants from across the multitude of publications, but have been limited by the utility due to the significant differences in approaches used to qualify pathogenicity of variants in each of the publications. The recent formulation of guidelines and algorithms for assessment of the pathogenicity of variants jointly put forward by the American College of Medical Genetics and the Association of Molecular Pathologists (ACMG &AMP) has provided a framework for evidence based and systematic assessment of pathogenicity of variants. In this paper, we describe a comprehensive resource of genetic variants in ATP7B gene manually curated from literature and data resources and systematically annotated using the ACMG & AMP guidelines for assessing pathogenicity. The resource therefore serves as a central point for clinicians and geneticists working on WD and to the best of our knowledge is the most comprehensive and only clinically annotated resource for WD. The resource is available at URL http://clingen.igib.res.in/WilsonGen/. We compiled a total of 3662 genetic variants from publications and databases associated with WD. Of these variants compiled, a total of 1458 were found to be unique entries. This is the largest WD database comprising 656 pathogenic/likely pathogenic variants reported classified according to ACMG & AMP guidelines. We also mapped all the pathogenic variants corresponding to ATP7B protein from literature and other databases. In addition, geographical origin and distribution of ATP7B pathogenic variants reported are also mapped in the database.

## Introduction

Wilson’s disease (WD) is an autosomal recessive mendelian disorder described by Kinnier Wilson in 1912. Until about 1948, the exact mechanism of the disease was not known. Cummings (1948) demonstrated that the disease is characterised by accumulation of Copper (Cu) in both liver as well as brain^1^. The prevalence of WD varies from 12 to 29 per 100,000 in European population^2^ and it is estimated that the global prevalence of WD is 1/10000 to 1/30000. It estimated that the carrier rate in the general population is approximately 1 in 90, making it one of the most prevalent mendelian disorders. The disease is caused by a dysfunctional ATP7B, a p-type ATPase protein which is involved in the metabolism of copper in the body. ATP7B is important for the incorporation of Cu in ceruloplasmin and excretion of the same in bile^3^. The defective gene results in a reduced excretion of Copper resulting in the accumulation of Cu in liver, central nervous system (CNS), cornea, kidney, joints and cardiac muscle^3^ which contribute to the characteristic clinical features of WD. The deposition of Cu mainly happens in the liver, which leads to many malfunctions and diseases such as hepatic dysfunction, hepatitis, haemolysis and cirrhosis^4,5^ Secondly, its deposition in the brain causes neurological implications like tremor, dystonia, dysgnosia, personality disorder, Parkinson like symptoms, bradykinesia and neuropsychiatric symptoms^6,7^. The Kayser-Fleischer (KF) ring, a rusty brown ring around the cornea is the single most important diagnostic sign in WD. It is found in 95% of patients belonging to neurowilson but it is not entirely specific for WD diagnosis^3,8^.

A number of studies have reported genetic variations in WD from across the world. In addition, some of the genetic variants are characterised by severe or mild phenotypes which makes molecular diagnosis an important part of the prognostication and management of the disease. A number of groups have previously attempted to create a comprehensive resource integrating data and evidence on WD. The major limitation in such an approach was the lack of a uniform system to annotate the pathogenicity of genetic variants for clinical interpretation, which precluded their widespread application in clinical settings. The recent guidelines on the annotation of genetic sequence variants put forward by the American College of Medical Genetics and the Association of Molecular Pathologists provides a uniform framework for systematic integration of evidence on each of the variants and classifies them based on the evidences obtained to infer their pathogenicity. In this manuscript, we describe how a systematic curation of genetic variants and systematic annotation of variants can fulfil the need for a clinically relevant resource for WD. To the best of our knowledge, this is the most comprehensive database of genetic variants in WD and the only resource with systematic classification of variants according to the ACMG and AMP guidelines.

## Materials and Methods

### Data curation

The ATP7B variants datasets available in the public domain were systematically compiled. A number of additional parameters were compiled for each of the variants in a systematic format. The parameters include (1) Genome Reference Consortium Human build 37 (GRCh37 / hg19) (2) references (available in our WD reporting engine) (3) HGVS NM (4) chromosome (5) AA change (6) reference gene (7) Pop (8) Gene Ori (9) Disease (10) OMIM ID (11) dbSNP (12) Population (13) HGVS NP (14) HGVS NM (15) HGVS NP (16) start (17) end (18) Reference base (19) Altered base (17) Homozygous/Heterozygous (18) Genetic Origin (19) technique (20) ethnicity (21) geographical origin (22) HGVS NG (23) HGVS NC (24) inheritance (25) ClinVarID (26) mutation (27) mutation effect.

The variants from the regional databases and journals published in English language were extracted and entry was made in spread excel sheet in accordance with the design format. This compilation encompassed a total of six datasets namely Wilson Disease Mutation Database University of Alberta, Neurodegenerative Diseases Variation Database (NDDVD), ClinVar, ATP7B mutations database (Universal Mutation Database, UMD), Leiden Open Variation Database (LOVD), Human Gene Mutation Database (HGMD) and publications. The variants are classified according to a joint consensus recommendation of the American College of Medical Genetics and Genomics and the Association for Molecular Pathology. All the datasets were based on the Human genome 19 (GRCh37/hg19) assembly. The variants were retrieved in variant call format from individual datasets and integrated into a master compendium using bespoke scripts in Python.

### LUMC Mutalyzer

This program is used to check sequence variation nomenclature based on the HGVS (Human Genome Variation Society) guidelines. The genomic, amino acid and coding position gaps were filled using the LUMC Mutalyzer tool^9^. It has many useful informative applications from where we used the position converter and syntax checker. The important pieces of information as mentioned above were extracted using position converter and syntax checker.

### Variant validator

This is an important tool for the validation and to aid HGVS nomenclature of variants, using this tool we validated all of variants retrieved from different sources^10^. The variants were manually checked and if errors were reported, the correction was performed with the help of UCSC browser. The variant calling format was retrieved using the variant validator and finally, annovar was used to annotate all variants of ATP7B gene.

### ACMG classification of ATP7B variants

The variants were re-classified as pathogenic, likely pathogenic, benign, likely benign and variants of uncertain significance (VUS) according to the ACMG guidelines^11^. The variants were processed in the first step based on allelic frequency, BA1 (>0.05), BS1 (0.01–0.05) and PM2 (<0.0005) as considered from three databases ExAC, 1000GP and ESP65000. In the second step, variants were assigned PP3 and BP4 based three computational databases (SIFT, PolyPhen2 and CADD) if at least two showed pathogenic and benign respectively. The third step was about to assign the pathogenic status for the PP5 and benign for the BP6, shreds of evidence based on the well annotated database as ClinVar. Along with this PM1 was assigned for the position of amino acid if it belongs to the most important domain of protein according to reference protein database Pfam^12^. And the fourth step dealt with literature mining, which includes 1. Functional assays (PS3; pathogenic and BS3; benign), 2. Control-Case studies (PS4; Odd Ratio >5 and BP5; if present in the control), 3. Cosegregation of variant in a gene with disease in multiple affected family members assigned as PP1, BS4; if there was no segregation reported in the literature, PS2; de novo variants confirmed in parents, PM6; de novo variant assumed in parents and PP4; considered if literature covering shows multiple family members with the disease symptoms cosegregated with variant and satisfying two more conditions (1, <50% benign variants present in the ClinVar for gene; 2, disorder must monogenic). The last step included the BP7 (same amino acid due to codon base substitution), PVS1 (if a variant fulfils 3 conditions were considered as PVS1; 1. change should be null variant, 2. should not be present in the last exon and last 50 base pairs of the penultimate exon, 3. Loss of function (LOF) of disease for the gene is a known mechanism), PS1 (at the same position, same amino change due to different base change in the same codon), PM5 (at the same position different amino acid), PM3 (compound heterozygous in the trans form), BP2 (compound heterozygous in the cis form), PM4 (Indels absent from the repeat region of particular gene) and BP3 (Indels present in the repeat region of particular gene). The pieces of evidence collected for the particular variant submitted to the Genetic Variant Interpretation Tool developed by the University of Maryland and interpretation of variant arranged in our database^13^.

### Database and web server

The Wilson Disease database was built as a web-based system for quick and easy access of variants. The variant data as well as the annotations were formatted to JavaScript Object Notation (JSON) and ported onto MongoDB v3.4.10. The web interface was coded in HTML5, CSS3, Bootstrap Material and AngularJS. CSS3, Bootstrap Material and AngularJS were used for tables and other visualization. The backend of this database was constructed using PHP 7.0, MongoDB v3.4.10 and the server was hosted using Apache HTTP server. MongoDB was used to keep track of data processing and resist the database through the web interface. AngularJS and PHP 7.0 scripts were used to retrieve the data from the database with search query.

## Results

**Figure 1:**
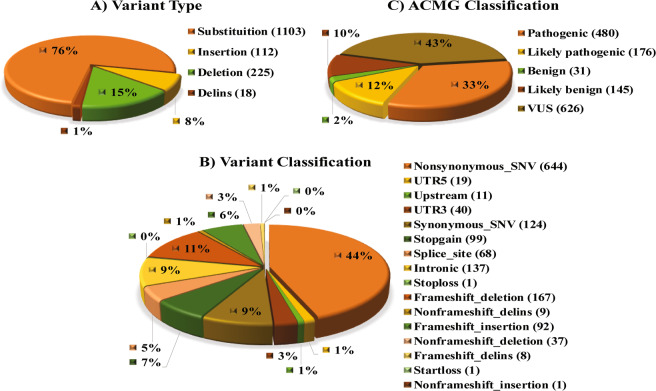
Classification of ATP7B variants as; (**A**) Variant type (**B**) Variant classification (**C**) ACMG Classification, based on ACMG guidelines, were classified as pathogenic, likely pathogenic, benign, likely benign and VUS (Variant of Uncertain Significance).

We compiled a total of 3662 genetic variants of ATP7B from publications and databases associated with WD (Supplementary Table 1). The variants compiled in the spreadsheet were observed in the exonic, splice site, intronic, 5 prime UTR, 3 prime UTR and upstream region of ATP7B gene and of which, 1458 were found to be unique. The unique variants based on the type of changes, summarised as substitution (1103), insertion (112), deletion (225) and delins (18) as explained in the Fig. 1A. Further based on the consequences of variants in the ATP7B gene and protein were classified in different classes as nonsynonymous, UTR5, upstream, UTR3, synonymous, stopgain, splice site, intronic, stop loss, frameshift deletion, non-frameshift delins, frameshift insertion, non-frameshift deletion, frameshift delins, start loss and non-frameshift insertion (Fig. 1B). These variants were systematically re-classified according to the ACMG & AMP standard guidelines in which 656 pathogenic/likely pathogenic, 176 benign/likely benign and 626 VUS. The distribution of the ACMG & AMP classified variants classes is summarized in Fig. 1C.

### Functional assay for Wilson’s disease

In context with functional analysis of ATP7B gene variants involved in the WD, is not well explored. Some variants had been studied in cell lines and yeast models and few assays are available in the scientific literature which we considered as one of the important defining criteria for pathogenicity of variants in the causation of disease. Literature survey shows many variants involved in the altered ATP7B gene/products activity (Supplementary Table 2).

### Frequency estimation

The variants, genomic annotations and pathogenicity assessment have been systematically compiled and made available as an online resource available at http://clingen.igib.res.in/WilsonGen/. The resource has been designed to be able to query the database using the variant genomic location or their pathogenicity classification. It also enables quick query of a batch of variants in the variant call format enabling clinicians and researchers to make quick assessments or genomic variants from panels/exomes or genomes. The reporting engine provides an easy to use interface to query variants annotation and pathogenicity in the database.

**Figure 2:**
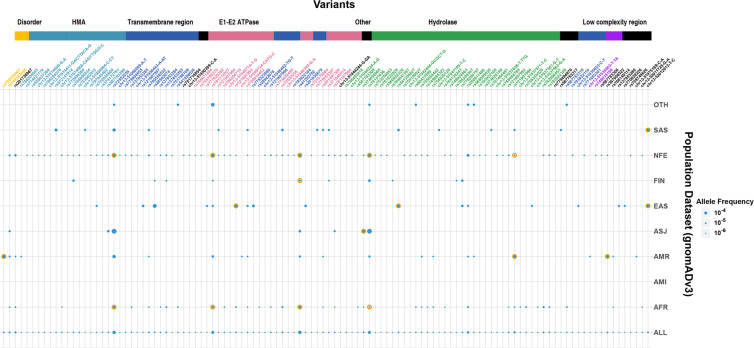
Frequency Estimation. Allele frequencies of the Pathogenic / Likely Pathogenic variants (656) and queried across the gnoMAD dataset of population genomes and exomes from across the world. 19 significant differences in allele frequency noted after Fisher’s Exact test and highlighted in yellow outer circles in the figure.

The availability of a well classified and annotated resource of genetic variants could also enable understanding of population specificity of genetic variants associated with WD. To this end, we systematically assessed the allele frequencies of the variants classified as pathogenic/likely pathogenic and queried across the gnoMAD dataset of population genomes and exomes from across the world. Our analysis suggests a number of variants show significant population enrichment Fisher’s Exact test (Corrected p value <0.05)) which would have implications in designing population scale screening and efficient molecular diagnostics for WD. These variants may also be used for the design of hotspots with respect to particular populations. The allele frequencies are summarized in Fig. 2.

### Distribution of pathogenic/likely pathogenic variants in ATP7B protein

**Figure 3:**
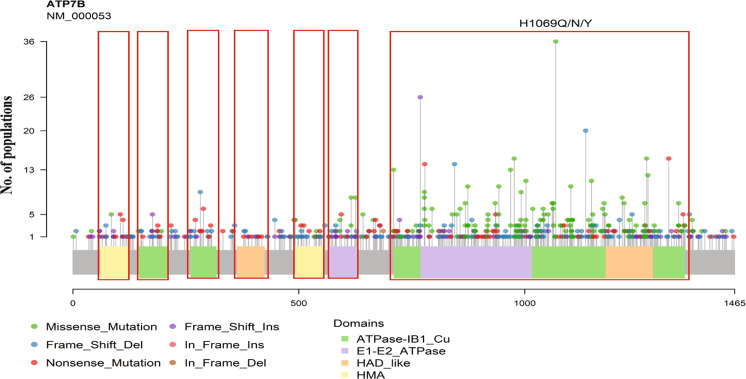
The depiction of the ACMG classified pathogenic and likely pathogenic variants on a ATP7B protein using lollipop plot.

**Figure 4:**
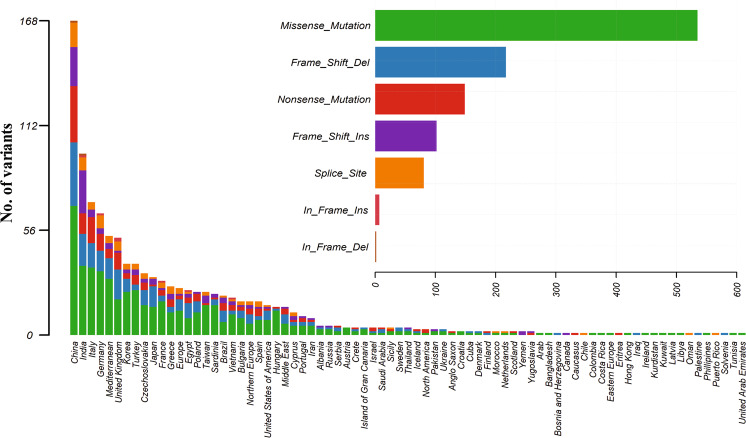
ATP7B pathogenic and likely pathogenic variant classes distribution at global scale.

**Figure 5:**
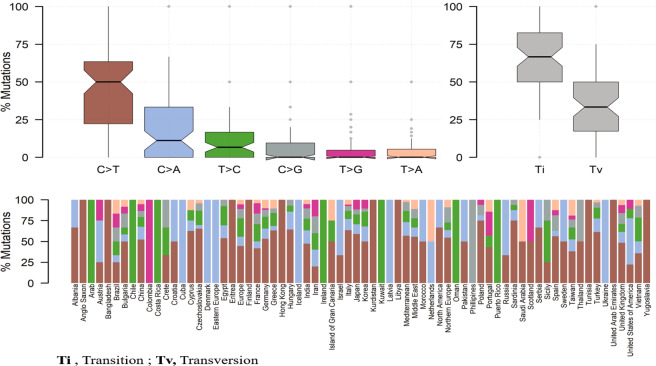
Depiction of pathogenic and likely pathogenic transition and transversion variants in ATP7B gene.

ATP7B gene consists of 21 exons and encodes a 158 kDa protein of 1465 amino acids length. This compendium of variants having 656 pathogenic and likely pathogenic variants in ATP7B gene of which 579 are present in the protein. To comprehend the significance of these protein variants in WD, we depicted these variants to the corresponding amino acid in ATP7B protein on the lollipop plot using reference transcript ID, NM_000053.3 as shown in Fig. 3. The most pathogenic and likely pathogenic variants were observed in the important functional domain of protein which might significantly affect the functionality of ATP7B protein. The height of lollipop shows distribution events in different populations, associated with pathogenicity of WD across the world populations have large heterogeneity in distribution (Fig. 4). We also depicted the pathogenic, likely pathogenic, transition and transversion variants in ATP7B gene also (Fig. 5)

#### Database and features

A user friendly WD variants search engine (WilsonGen) was designed that can be publicly accessed through URL http://clingen.igib.res.in/WilsonGen/. The search data tool is used to find variants reported in ATP7B till Feb., 2019. The pieces of information of variant can be accessed by assigning the following search queries; variant genomic position, amino acid position, gene name, genomic region and dbSNP ID. Example; Table 1

**Table 1:** WD variants search engine (WilsonGen).

Variant	13-52520526-C-T, 13:52518390:G:A
Gene	ATP
Amino acid change	R952K, S693Y, W939C
Region	13-52532633-52532646, 13-52516569-52516568, 13-52516633
dbSNP ID	rs121907994, rs732774

## Discussion

A comprehensive well curated and clinically annotated genetic variant resource is considered as a starting point to clinical diagnosis as well understanding the genetics, epidemiology, genotype phenotype correlation and accurate prognostication of the disease. It is also noteworthy to mention that the resource would also be a central point to enable evidence-based genetic counselling of patients. The recent availability of a comprehensive and evidence based guideline and algorithm for classification of the pathogenicity of genetic variants put across by the ACMG & AMP along with initiatives to systematically annotate variants provide a unique opportunity to put together a precision medicine through genetic testing. It should also be emphasized that the comprehensive resource along with the genetic epidemiology would serve as a starting point to developing cost effective tests for case identification as well as carrier screening with an emphasis on early diagnosis, prevention and genetic counselling. WilsonGen tries to fill in the gap and in future extensively work towards being a central resource for collecting and disseminating genetic evidence on WD.

## Supplementary information

Supplementary information.
