# Supplementary material for: WilsonGen a comprehensive clinically annotated genomic variant resource for Wilson’s Disease
Source: Sci Rep. 2020 Jun 3;10:9037. doi: 10.1038/s41598-020-66099-2 (PMC7270127; doi:10.1038/s41598-020-66099-2)
Supplement: Supplementary file 1 — Supplementary information. [file 41598_2020_66099_MOESM1_ESM.docx]

**Supplementary Table 1:** Overview of variants reported in ATP7B gene

**Supplementary Table 2:** Literature survey shows many variants involved in the altered ATP7B gene/products activity.

**Supplementary Table 1**

**WilsonGen a comprehensive clinically annotated genomic variant resource for Wilson’s Disease**

**Mukesh Kumar^1,2^, Utkarsh Gaharwar^1^, Sangita Paul^1^, Mukta Poojary^1,2^, Kavita Pandhare^1^, Vinod Scaria^1,2*^ and Binukumar BK^1,2*^**

***Correspondence to: 1. Dr.Vinod Scaria*, Senior Scientist, CSIR-Institute of Genomics and Integrative Biology (IGIB), Mathura Road, Sukhdev Vihar, New Delhi 110025 India.*** [***vinods@igib.in***](mailto:vinods@igib.in)***. 2. Dr. Binukumar BK*, Senior Scientist, CSIR-Institute of Genomics and Integrative Biology (IGIB), Mall Road, New Delhi 110007, India. binukumar@igib.in***

| **Variants Sources** | **Curators** | **No of variants** | **Last updated** |
| --- | --- | --- | --- |
| Wilson Disease Mutation Database, University of Alberta | Diane Cox, Lisa Davies, Dept. Med Gen University of Alberta, Canada | 1184 | stopped in 2010 |
| Universal Mutation Database (UMD) | Irene Ceballos, Paris, France | 327 | 4/13/2006 |
| Global Variome, LOVD 3.X | LOVD-team | 225 | 12/14/2018 |
| Leiden Open Variation Database, LOVD 3.X | Graham Taylor  University of Melbourne |  | 6/12/2013 |
| BIPMed SNP Array, LOVD 3.X | Admin BRAINN |  | 10/11/2018 |
| BIPMed WES, LOVD 3.X | Admin BRAINN |  | 12/19/2018 |
| Neurodegenerative Diseases Variation Database (NDDVD), LOVD 3.X | Y Yang  SUDA | 51 | 6/29/2017 |
| Human Gene Mutation Database (HGMD) and reference articles | P.D. Stenson | 681 | 2017 |
| ClinVar | - | 545 | 4/3/2019 |
| Research articles published in English language | - | 649 | May, 2019 |
| Total variants | - | 3662 | - |

**Supplementary Table 2**

**WilsonGen a comprehensive clinically annotated genomic variant resource for Wilson’s Disease**

**Mukesh Kumar^1,2^, Utkarsh Gaharwar^1^, Sangita Paul^1^, Mukta Poojary^1,2^, Kavita Pandhare^1^, Vinod Scaria^1,2*^ and Binukumar BK^1,2*^**

***Correspondence to: 1. Dr.Vinod Scaria*, Senior Scientist, Center for Genome Informatics, CSIR-Institute of Genomics and Integrative Biology (IGIB), Mathura Road, Sukhdev Vihar, New Delhi 110025 India. vinods@igib.in***

***2. Dr. Binukumar BK*, Senior Scientist, CSIR-Institute of Genomics and Integrative Biology (IGIB), Mall Road, New Delhi 110007, India.*** [***binukumar@igib.in***](mailto:binukumar@igib.in)

| **Variants** | **Model** | **Techniques/Methods** | **Activity of ATP7B gene/ product** | **References** |
| --- | --- | --- | --- | --- |
| D1027A, G85V, L492S, G1266R, E1064K, D1222V, R616W, G626A, G710S, P760L, P840L, R969Q, T1031S, P1052L, H1069Q, N1270S, P1273L, S1362Ffs, A874V and L1083F | Sf9 cells and HEK293 T-REx cells | Tet-on/off system in HEK293T cells | Affected | PMID: 22240481 |
| R616Q, G85V, Y741C, L745R, A874V, I1230T and R1301Q | Ccc2-deficient S. cerevisiae strain BY4741 | Gene Therapy/complementation | Affected | PMID: 30702195 |
| R788L | Hepatocyte-like cells from Wilson's disease iPSC | Gene Therapy/complementation | Affected | PMID: 21593220 |
| R778Q, R778L and P992L | FET3 and Ccc2 deficient S. cerevisiae strain BJ2168 | Gene Therapy/complementation | Affected | PMID: 9837819 |
| T788I, V1036I and R1038G-fsX83 | Ccc2-deficient yeast cells and HepG2 cell line | Gene Therapy/complementation | Affected | PMID: 26004889 |
| D1027A, TGE858‐860AAA, C1104F, G1341V LLL1454‐1456AAA, CPC983‐985SPS, G85V, R778L, H1069Q, and V1262F | Human osteosarcoma cell line (U2OS) and HEK293T cells | Gene Therapy/complementation | Affected | PMID: 19937698 |
| G875R | HEK293TREx cells and YSTT cells transfected with tyrosinase | Gene therapy in HEK293TREx cells and Copper deposition in YSTT cells by checking activity of transfected tyrosinase | Affected | PMID: 21406592 |
| R778L and P992L | CHO cell line | Gene Therapy/complementation | Affected | PMID: 26032686 |
| S986F, I1348N, G1355D, M1392K, and A1445P, 2810delT, -133A>C and -215A>T | CHO‐K1 cells | The viability of ATP7B‐transfected CHO‐K1 cells in the presence of different concentrations of copper | Affected | PMID: 20931554 |
| T991M, G1000R, R1228T, L1043P, G1101R, I1102T, V1239G, D1267V and G1287S | BJ2168 Ccc2-deficient yeast | Gene Therapy/complementation | Affected | PMID: 20333758 |
| E1064K, V1106D, L1083F and M1169V | Ccc2-deficient yeast | Gene Therapy/complementation | Affected | PMID: 18203200 |
| H1069Q and N1270S | Ccc2-deficient Saccha-romyces cerevisiae | Gene Therapy/complementation | Affected | PMID: 9654149 |
| C1091Y, T1029I, R778L, M729V, G891D, V1024A, T1031A and C656X | Ccc2-deficient Saccha-romyces cerevisiae | Gene Therapy/complementation | Affected | PMID: 17587212 |
| c.2487dupT, H1069Q, N1270S and 2304dupC | Lymphoblastoid cell lines derived from patients and control | Comparative in-vitro analysis of ATPase activity of cell membrane extracted from LCL derived from patients and control | Affected | PMID: 9311736 |
| V1262F | Human osteosarcoma cell line (U2OS) and HEK293T cells | Gene Therapy/complementation | Affected | PMID: 19937698 |
| L1373P and L1373R | SV40-transformed Menkes-null fibroblasts (YS and YSTT) and WIF-B Cells | Gene Therapy/complementation | Affected | PMID: 21454443 |
| C305X | HEK293T | Gene Therapy/complementation | Affected | PMID: 30723317 |
| E332X, Q511X, Q547X and Q819X | Chinese hamster ovary (CHO) and SH-SY5Y cells | Gene Therapy/complementation | Affected | PMID: 23607698 |
| -133A>C and -215A>T | CHO cell line | Promoter assay/Reporter Gene Assay | Affected | PMID: 20931554 |
| P534T, P539H, D642Y, R723T, R725T and A727D | BxPC3, AsPC1, HeLa, NCI-H358, and CHO cell lines (Negligible ATP7B expression) | Comparative study of ATP7B variants in BxPC3, AsPC1, HeLa, NCI-H358, and CHO cell lines (Negligible ATP7B expression by Western blotting, Cell viability assay, Intracellular copper accumulation, Copper excretion in media and Ceruloplasmin secretion in media | Affected | PMID: 30120852 |
| E122fs, M573fs, C271X, R778L | HepG2 ATP7B Knockout (KO) cell line | Gene Therapy/complementation | Affected | PMID: 27122662 |
| N41S | WIF-B Cells | Gene Therapy/complementation | Affected | PMID: 19033537 |
| G85V and G591D | HEK293T and HepG2 | pEBB-ATP7B-Flag tagged plasmid transfection | Affected | PMID: 17919502 |
